# Dysregulation of a Heme Oxygenase–Synuclein Axis in Parkinson Disease

**DOI:** 10.3390/neurosci3020020

**Published:** 2022-05-20

**Authors:** Marisa Cressatti, Hyman M. Schipper

**Affiliations:** 1 Integrated Program in Neuroscience, McGill University, Montreal, QC H3T1E2, Canada; marisa.cressatti@mail.mcgill.ca; 2 Lady Davis Institute for Medical Research, Jewish General Hospital, Department of Neurology & Neurosurgery, McGill University, 3999 Cote Sainte-Catherine Road, Montreal, QC H3T1E2, Canada

**Keywords:** α-synuclein, biomarker, heme oxygenase-1, microRNA, Parkinson disease, transgenic mouse

## Abstract

α-Synuclein is a key driver of the pathogenesis of Parkinson disease (PD). Heme oxygenase-1 (HO-1), a stress protein that catalyzes the conversion of heme to biliverdin, carbon monoxide and free ferrous iron, is elevated in PD-affected neural tissues and promotes iron deposition and mitochondrial dysfunction in models of the disease, pathways also impacted by α-synuclein. Elevated expression of human HO-1 in astrocytes of GFAP.HMOX1 transgenic mice between 8.5 and 19 months of age elicits a parkinsonian phenotype characterized by nigrostriatal hypodopaminergia, locomotor incoordination and overproduction of neurotoxic native S129-phospho-α-synuclein. Two microRNAs (miRNA) known to regulate α-synuclein, miR-153 and miR-223, are significantly decreased in the basal ganglia of GFAP.HMOX1 mice. Serum concentrations of both miRNAs progressively decline in wild-type (WT) and GFAP.HMOX1 mice between 11 and 18 months of age. Moreover, circulating levels of miR-153 and miR-223 are significantly lower, and erythrocyte α-synuclein concentrations are increased, in GFAP.HMOX1 mice relative to WT values. MiR-153 and miR-223 are similarly decreased in the saliva of PD patients compared to healthy controls. Upregulation of glial HO-1 may promote parkinsonism by suppressing miR-153 and miR-223, which, in turn, enhance production of neurotoxic α-synuclein. The aim of the current review is to explore the link between HO-1, α-synuclein and PD, evaluating evidence derived from our laboratory and others. HO-1, miR-153 and miR-223 and α-synuclein may serve as potential biomarkers and targets for disease-modifying therapy in idiopathic PD.

## 1. Introduction

The neurodegenerative movement disorder, Parkinson disease (PD) is the most prevalent of several human synucleinopathies. Shortly after the discovery of SNCA mutations causing a rare monogenic form of PD in 1997 [1], α-synuclein protein aggregates were identified as a major component of hallmark Lewy bodies and a key player in PD pathology [2]. As with other synucleinopathies, including multiple system atrophy and Lewy body dementia, cardinal symptoms of PD include bradykinesia, rigidity, rest tremor and postural instability. Non-motor symptoms, such as cognitive decline, hyposmia (loss of sense of smell), rapid eye movement (REM) sleep behavior disorder (RBD), constipation and other autonomic dysfunctions and depression and anxiety, complete the clinical picture. Although symptomatic pharmacotherapy is available, there currently exists no treatment that unequivocally mitigates neuronal attrition and clinical decline in this condition.

## 2. α-Synuclein: A Key Player in Parkinson Disease

α-Synuclein has long been considered a major component of PD pathology. While precise mechanisms of abnormal α-synuclein aggregation remain disputed, there is fair consensus implicating oxidative reactions in this process [3,4]. Dimerization of Tyr125 is the initial and rate-limiting step that ultimately leads to a greater potential for self-interaction of this protein [5]. The dimerized α-synuclein serves as the template for native α-synuclein monomers to refold into oligomers, protofibrils and fibrils rich in β-pleated sheets. α-Synuclein oligomers, protofibrils and fibrils contribute to neuronal death via oxidative stress, energy failure, excitotoxicity and neuroinflammation [6,7]. Phosphorylation, oxidation, nitration and glycation are post-translational modifications of α-synuclein that enhance its propensity for aggregation [8]. Of particular importance, phosphorylation is a necessary event during the formation of Lewy bodies, as de-phosphorylation, specifically at Ser129, ameliorates the phenotype [9]. These are important considerations when evaluating α-synuclein as a potential biomarker for neurodegenerative synucleinopathies, such as PD.

α-Synuclein has been the primary focus of many PD biomarker studies. Altered α-synuclein levels (soluble, aggregated and post-translationally modified forms) in cerebrospinal fluid (CSF), blood and saliva have been reported, with general consensus describing reduced total α-synuclein, higher oligomeric α-synuclein and higher oligomeric-to-total-α-synuclein ratios in PD patients compared to controls [10,11,12,13,14,15,16,17,18]. Relating to α-synuclein but acting upstream, we have also reported alterations in circulating levels of potential α-synuclein regulators, including heme oxygenase-1 (HO-1), microRNA (miR)-153 and miR-223 [19,20,21].

The current review is centered on the link between α-synuclein, HO-1 and key miRNAs as they relate to PD.

## 3. Involvement of Heme Oxygenase-1 in Parkinson Disease Pathology

**Figure 1:**
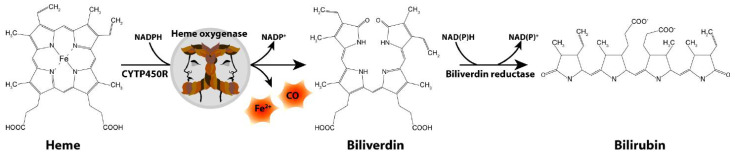
Heme degradation pathway. CO, carbon monoxide; CYTP450R, cytochrome P450 reductase; Fe^2+^, ferrous iron.

The Schipper laboratory has been studying neurodegenerative disorders, such as PD, for over two decades through the lens of a highly inducible stress protein, HO-1 [22]. HO-1 catalyzes the conversion of heme into biliverdin, carbon monoxide and free ferrous iron in brain and other tissues (Figure 1). The *HMOX1* promoter contains numerous response elements which render the gene exquisitely sensitive to induction by heme, β-amyloid, lipopolysaccharide, hyperoxia, ultraviolet light, IL-1β, TNF-α, hydrogen peroxide and heavy metals, among many others [23]. Though typically assuming a protective role via the antioxidant properties of biliverdin and bilirubin [24,25,26,27], our laboratory and others have shown that chronic overexposure of HO-1 may exacerbate intracellular oxidative damage in mitochondria and other subcellular compartments via heme-derived iron and carbon monoxide [28,29,30,31]. The latter may prevail in PD and other chronic neurodegenerative conditions.

Astrocytes and microglia in PD basal ganglia, although not neurons, exhibit significant elevations of HO-1 protein [32,33]. *HMOX1* induction in dopaminergic neurons is commensurate with the fact that, during normal aging, nigral dopaminergic neurons are subjected to elevated levels of reactive oxygen species (ROS) arising from the iron-catalyzed oxidation and enzymatic deamination of dopamine [34]. Further, cytoplasmic Lewy bodies within affected dopaminergic neurons of the PD substantia nigra exhibited prominent HO-1 immunoreactivity [32,35]. Some studies have linked polymorphisms in *HMOX1* to the disease. One such study disclosed a strong association between rs2071746 (A/T), a single-nucleotide polymorphism that enhances *HMOX1* promoter activity/transcription and clinical idiopathic PD [36]. Another group found a synergistic association of *HMOX1* rs2077146TT polymorphism and pesticide exposure, specifically increasing the risk for PD [37]. Finally, several polymorphisms in the promotor region of *NRF2* and the Nrf2 binding region of the microtubule-associated protein tau (*MAPT*) promoter, a known transcriptional activator of *HMOX1*, have been associated with PD [38,39].

Astrocyte activation, particularly in the substantia nigra, has been documented in patients with PD and animal models of PD [40,41]. Astrocyte activation can produce TNF-α, IL-1β, nitric oxide and other inflammatory factors, all of which may lead to the induction of *HMOX1*. In addition to pro-inflammatory cytokines, other plausible inducers of *HMOX1* in astrocytes of the PD substantia nigra include environmental or endogenous MPTP-like neurotoxins and dopamine-derived ROS [42,43]. The local uptick in glial HO-1 activity seen in PD may accelerate the deposition of non-transferrin iron and mitochondrial complex I deficits documented in PD-affected neural tissues [44]. This glial mitochondrial iron mediates the oxidation of dopamine to neurotoxic o-quinone radicals [44] and oxidizes the pro-neurotoxin MPTP to the dopamine neurotoxin MPP+ [45], which inhibits mitochondrial complex 1 and leads to oxidative stress, the loss of adenosine triphosphate, protein nitration and apoptosis of dopaminergic neurons [41,46]. Autophagy in astrocytes is considered to have a vital role in the pathogenesis of aging and neurodegenerative disease, particularly for the removal of pro-oxidant species [47]. Furthermore, brain astrocytes exposed to high glucose concentration undergo apoptosis through the activation of HO-1 via NF-kB and AP-1, providing evidence for Nrf2-independent induction of neurotoxic HO-1 [48]. In addition to the above reports, extensive in vitro and in vivo evidence from our laboratory and others has directly implicated HO-1 in PD pathogenesis [22].

*HMOX1* transfection of human M17 neuroblastoma was shown to stimulate proteasomal catabolism of WT α-synuclein [49] but had no effect on proteasomal degradation of A30P α-synuclein, a mutant protein implicated in familial autosomal-dominant PD [1]. Insofar as non-digestible α-synuclein aggregates are cytotoxic [50], the imperviousness of A30P α-synuclein protein to HO-1-directed proteolysis may contribute to Lewy body formation and parkinsonism in people with this mutation [49]. Iron is also believed to serve as a major contributor by inducing oxidative stress and α-synuclein aggregation, and changes in Nrf2 and HO-1 have been shown contribute to iron-induced α-synuclein aggregation [51]. Xu et al. showed a significant positive correlation between elevated serum HO-1 concentrations and iron deposition within the substantia nigra and an inverse correlation between elevated serum HO-1 and reduced hemoglobin levels in PD patients relative to controls [52]. This increase in HO-1 may be a common mechanism underlying the iron deposition and low hemoglobin documented in PD. Another study demonstrated a time-dependent HO-1 upregulation in primary cultured ventral mesencephalon astrocytes treated with MPP+ or recombinant α-synuclein [53]. Attesting to the dual nature of HO-1, treatment with cobalt protoporphyein IX, an HO-1 activator, exerted protective effects against MPP+ or α-synuclein during moderate HO-1 upregulation, but it aggravated damage at the peak of the HO-1 response [53]. Importantly, core cytopathology implicated in PD (oxidative stress, mitochondrial damage and macroautophagy) is abrogated in *HMOX1*-transfected primary astrocytes treated with the competitive HO inhibitor, tin mesoporphyrin [54].

This in vitro work led to the development of a novel transgenic mouse model, the GFAP.HMOX1^8.5−19 m^ mouse, in which human *HMOX1* was selectively overexpressed in astrocytes between 8.5 to 19 months of age [55]. The transgene cascade of the GFAP.HMOX1 mouse leads to activation of the human HO-1 coding sequence through the upstream promoter drive of glial fibrillary acidic protein (GFAP) and the “valve-controller” of tetracycline activator. This design confers two advantages: (1) The GFAP promoter selectively targets *HMOX1* gene expression to the astrocytes, and (2) the Tetracycline (Tet)-Off system permits temporal control of the transgene expression [55]. The doxycycline diet, which inhibits transgene expression, was replaced with a regular rodent diet between 8.5 and 19 months of age to permit *HMOX1* expression in astrocytes [55]. Our glia-centric focus on HO-1 expression is pivotal to our group’s longstanding perspective on mechanisms of neurodegeneration in the parkinsonian brain. This approach is based on the following considerations: (1) The excess iron reported in the aging and PD brain largely implicates glial and other non-neuronal cells [23]. (2) HO-1 is significantly upregulated in the astrocytes, not neurons, of the PD substantia nigra relative to normal age-matched control values [32]. Further, astrocytes and microglia have a greater propensity to mount a strong HO-1 (as well as other stress protein) response compared to neurons and oligodendrocytes [56,57,58]. (3) *Hmox1* induction by stressors implicated in PD (e.g., hydrogen peroxide, heavy metals, TNF-α, etc.) is a common pathway leading to mitochondrial iron deposition, oxidative mitochondrial damage and macroautophagy in aging subcortical astroglia [23,31,32,59]. (4) The ability of astrocytes to effectively revert to anaerobic metabolism for their energy needs, also known as the Warburg effect, may permit astroglia (but not neurons) to sacrifice a considerable fraction of their mitochondria with minimal consequence [43]. (5) Finally, the progressive accumulation of glial-mitochondrial iron within subcortical brain regions enhances the vulnerability of nearby dopaminergic neurons to oxidative injury and may thereby render the senescent central nervous system (CNS) more prone to PD [44,59]. For these reasons, *HMOX1* was selectively overexpressed in *astrocytes* of this novel transgenic mouse model. Adding further predictive validity to our model, inhibition of HO-1 activity in vitro or in vivo rescues the resulting phenotype [54,60]. The following section outlines evidence accrued from parkinsonian GFAP.HMOX1^8.5−19 m^ mice.

## 4. GFAP.HMOX1^8.5−19 m^ Transgenic Mice

At 19 months of age, GFAP.HMOX1^8.5−19 m^ mice recapitulate key features of PD relative to age-matched wild-type (WT) controls, as evidenced by the following: (i) impaired locomotion (rotarod), circling behavior, motor incoordination (pole test), altered ambulation (gait test) and reduced olfaction (buried pellet test); (ii) dopaminergic neuron degeneration in the substantia nigra, with substantial vacuolation manifesting in the remaining neurons and decreased dopamine levels in the striatum; (iii) upregulated pituitary homeobox 3 (Pitx3)– and dopamine transporter (DAT)–targeting miR-133b and nuclear receptor related-1 protein (Nurr1)–targeting miR-145 in the nigrostriatum; (iv) significant elevation of gamma amino butyric acid (GABA) in the substantia nigra [61,62,63]; (v) increased iron deposition and improved rotarod performance after treatment with the iron chelator deferiprone; (vi) enhanced protein carbonylation, a marker of oxidative stress, in the striatum; (vii) reduced and disorganized mitochondrial cristae and fragmented mitochondrial membranes within the striatum [55,64,65]; and (viii) dysregulated autophagy and accumulation of striatal osmiophilic inclusions, indices of autophagosome formation and mitophagy. Notably, this phenotype was not observed in mice expressing the *HMOX1* transgene for an identical duration between 1.5 and 12 months of age [55], underscoring the importance of brain aging for symptom manifestation in both human and experimental parkinsonism.

The upregulation of brain HO-1 is similarly observed in other animal models of PD, including MPTP- and rotenone-treated mice [66,67,68]. HO-1 upregulation was also associated with loss of dopaminergic neurons in the MPP+-induced parkinsonian rat model [69]. Furthermore, the significant increases in serum HO-1 levels and iron deposition in the substantia nigra observed in the MPTP mouse model of PD were abrogated by treatment with the HO inhibitor tin mesoporphyrin [52]. HO-1 is involved in a number of other disease pathways, primarily due to the high inducibility and ubiquity of *HMOX1*. Complete *Hmox1* knockout results in a general pro-inflammatory phenotype in mice [70,71], attesting to the protective nature of heme degradation products, biliverdin and bilirubin. The cellular localization of HO-1 also seems to be important. While we and others report a behavioral, pathological and biochemical phenotype consistent with PD when *HMOX1* overexpression is restricted to astrocytes, overexpression of HO-1 in neurons in mice protects against oxidative insult [72]. Transgenic mice overexpressing HO-1 in neurons also exhibit low levels of lipid peroxidation end-products and enhanced expression of the anti-apoptotic protein bcl-2 after cerebral ischemia [73]. Similarly, upregulation of HO-1 in microglia confers a strong anti-inflammatory effect and reduction in oxidative damage [74,75]. The chronicity of HO-1 action appears to be a crucial factor in determining outcome as *HMOX1* in the GFAP.HMOX1 mouse model confers significant neuroprotection in the face of acute intracerebral hemorrhage [76,77].

### 4.1. GFAP.HMOX1^8.5–19 m^ Mice and α-Synuclein

**Figure 2:**
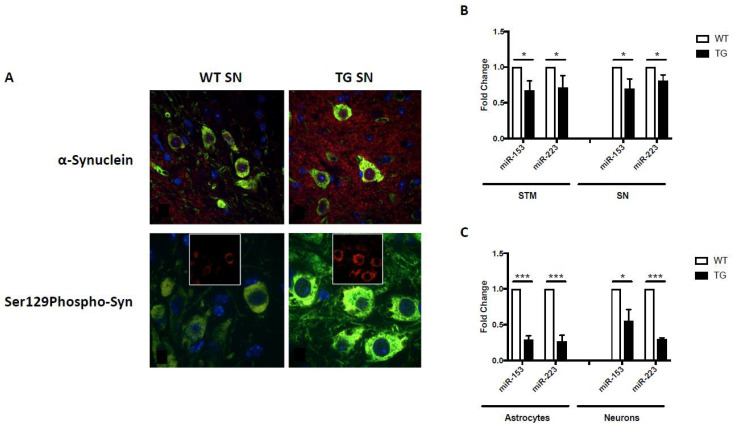
α-Synuclein expression and miRNA regulation in GFAP.HMOX1 mice. (**A**) Confocal imaging of intra- and extraperikaryal α-synuclein (red) and Ser129-phosphorylated (S129Phospho) α-synuclein (red) immunofluorescence in dopaminergic neurons of the substantia nigra (SN). TH (green) and DAPI (blue) in 19-month-old WT and transgenic substantia nigra. (**B**) Downregulation of miR-153 and miR-223 in transgenic substantia nigra (SN) and striatum (STM) in vivo and (**C**) transgenic primary astrocyte–neuronal co-cultures. All data were obtained via RT-qPCR and analyzed using the ΔΔCt method relative to internal and endogenous controls. *n* = 5; * *p* < 0.05; *** *p* < 0.001. (From [64], with permission).

In addition to the aforementioned behavioral, pathological and biochemical alterations, GFAP.HMOX1^8.5−19 m^ mice also exhibited significant α-synuclein-related pathologies. Notably, α-synuclein and pathological Ser129-phosphorylated α-synuclein were significantly increased within and surrounding the dopaminergic neurons of the substantia nigra of 19-month-old GFAP.HMOX1^8.5−19 m^ mice compared to age-matched WT controls (Figure 2A) [55,64]. Significant elevations in ubiquitin, ubiquitin-binding protein p62 (p62) and HO-1, previously associated with Lewy body inclusions [32,78,79], were also observed in our transgenic mice, though no overt Lewy pathology was noted [55]. The lack of Lewy body inclusions observed in 19-month-old GFAP.HMOX1^8.5−19 m^ mice, a common criticism of parkinsonian animal models, may be rationalized as follows: (i) GFAP.HMOX1^8.5−19m^ mice may represent a mid-stage model of PD, considering the other early-to-mid-stage features of parkinsonism in this model, such as reduced olfaction and only 47% loss of nigral dopaminergic neurons [55,65]; whether GFAP.HMOX1^8.5−19 m^ mice would display Lewy body inclusions later in life (e.g., at 24 months of age) remains to be determined; and (ii) *HMOX1* overexpression may not be sufficient to elicit Lewy body inclusion formation. To interrogate the latter, offspring of GFAP.HMOX1 mice crossed with an α-synuclein overexpressing model could be assayed for augmented Lewy body inclusion formation. Enhanced α-synuclein production and aggregation has been linked to increased levels of iron, and induction of *HMOX1* was noted to result in greater α-synuclein protein deposition in vitro [64]. Further supporting the link between HO-1 and α-synuclein, *HMOX1* overexpression from embryogenesis until 12 months of age, as seen in the GFAP.HMOX1^0–12 m^ mouse model of schizophrenia, similarly resulted in significant elevation of α-synuclein mRNA and protein [80]. 

### 4.2. GFAP.HMOX1 Primary Cultures and α-Synuclein

Targeting α-synuclein is an important therapeutic consideration in PD, highlighted by the fact that siRNA inhibition of the gene encoding this protein, *Snca*, in primary GFAP.HMOX1 astrocytes attenuated oxidative stress to levels observed in negative control and WT preparations [64]. Moreover, when co-culturing primary GFAP.HMOX1 astrocytes with WT neurons, followed by treatment with siRNA against *Snca*, WT neurons displayed normalization of mRNAs involved in oxidative stress (MnSOD), dopaminergic neuron development and maintenance (Nurr1, Pitx3), dopamine metabolism (TH, DAT), mitophagy (DJ-1), mitochondrial fission (Drp1) and mitochondrial fusion (Mfn2, Mfn1) [64]. These observations underscore the importance of α-synuclein suppression in mitigating key pathomechanisms in PD, possibly in combination with upstream HO-1 inhibition. Potentially viable targets of α-synuclein mitigation include miRNAs, miR-153 and miR-223, which were found to negatively regulate α-synuclein mRNA and protein [64]. 

### 4.3. MicroRNAs In Vitro and In Vivo

MiRNAs have gained immense traction as key regulators in development, normal aging and disease, including PD [81,82,83]. In 2015, we published a study looking at miRNA profiles in *HMOX1*-transfected primary rat astrocytes compared to sham-transfected controls and identified three significantly upregulated miRNAs (miR-140*, miR-17 and miR-16) and six significantly downregulated miRNAs (miR-297, miR-206, miR-187, miR-181a, miR-138 and miR-29c) in the *HMOX1*-transfected cells [84]. Moreover, the effects of HO-1 induction on glial miRNA profiles were abrogated by a competitive HO inhibitor (tin mesoporphyrin), an iron chelator (deferoxamine) and a CO antagonist (methylene blue), directly implicating HO-1, iron and CO, respectively, in the aberrant miRNA expression profiles [84]. On the other hand, the addition of bilirubin, the final product of heme catabolism, had little effect on these miRNA levels in cultured astrocytes [84]. These salient miRNA profiles were recapitulated in WT cells treated with the iron donor Fe(NO_3_)_3_ [84]. Taken together, these results lend support to the canonical nature of HO-1 activity in our system and its role in the perturbed expression of PD-relevant miRNAs.

Akin to miRNA changes in *HMOX1*-transfected primary rat astrocytes, GFAP.HMOX1^8.5−19 m^ mice and primary GFAP.HMOX1 astrocyte–neuron co-cultures display altered miRNA expression profiles. In addition to changes in Pitx3- and DAT-targeting miR-133b and Nurr1-targeting miR-145 described above [55,64], changes in miRNAs targeting α-synuclein were also observed. Conserved binding sites for miR-153 and miR-223 were predicted to lie within the 3′-untranslated region (UTR) of *SNCA* with partial complementarity (TargetScan software), and miR-153 was confirmed to bind the 3′-UTR of *SNCA* and suppress α-synuclein mRNA and protein [85,86,87]. This partial complementarity suggests regulation at the level of both SNCA mRNA via targeted mRNA degradation and α-synuclein protein via translation repression, a notion that is supported by our mimic and inhibitor experiments [64]. M17 human neuroblastoma cells transfected for 12 h with either a miR-153 or miR-223 mimic resulted in significant downregulation of α-synuclein mRNA and protein relative to controls [64]. Conversely, M17 human neuroblastoma cells transfected for 12 h with either a miR-153 or miR-223 inhibitor resulted in significant upregulation of α-synuclein mRNA and protein relative to controls [64]. In the earlier *HMOX1*-transfected primary rat astrocyte experiments, miR-153 expression trended downwards while miR-223 showed no significant change, though these results were not further validated by RT-qPCR [84]. In GFAP.HMOX1^8.5−19 m^ mice; on the other hand, α-synuclein-targeting miR-153 and miR-223 were significantly downregulated in the substantia nigra and striatum, as well as in primary astrocyte and neuron co-cultures generated from transgenic mouse brain, compared to WT control preparations (Figure 2B,C) [64]. This falls in line with the apparent upregulation of α-synuclein protein observed in GFAP.HMOX1^8.5−19 m^ mouse brains [64] and further attests to the link between HO-1, α-synuclein and miR-153/miR-223.

Other targets of miR-153 include β-amyloid and nuclear factor erythroid 2-related factor 2 (Nrf2). Similar to α-synuclein deposition in the PD brain, suppression of miR-153 has been correlated with high levels of amyloid precursor protein and β-amyloid in the Alzheimer disease (AD) brain [88,89]. Additionally, inhibition of miR-153 promotes the expression of Nrf2 and HO-1 [90], and this miRNA has been reported to be dysregulated in the APPswe/PS1_ΔE9_ mouse model of AD [91] as well as the MPTP mouse model of PD [92]. Other targets of miR-223 include NF-kB and IL-1. Notably, β-amyloid, amyloid precursor protein, Nrf2, NF-kB and IL-1 have all been identified as inducers of *HMOX1* [22].

**Figure 3:**
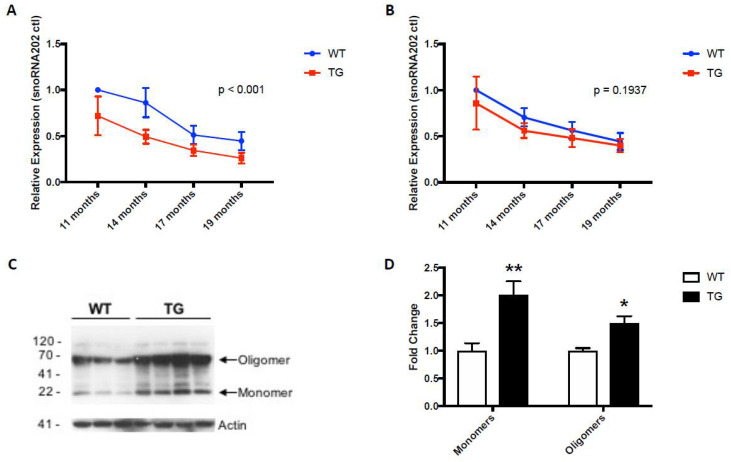
Expression patterns of miR-153 (**A**), miR-223 (**B**) and α-synuclein (**C**,**D**) in WT and GFAP.HMOX1 mouse blood. Blood serum was procured from WT and transgenic mice at 11, 14, 17 and 19 months of age, while RBCs were isolated from WT and transgenic mice at 19 months of age only. MiRNA expression levels were determined by RT-qPCR and analyzed using the ΔΔCt method relative to internal and endogenous controls. Western blot was used to measure α-synuclein protein. For (**A**,**B**), *n* = 7–13; for (**C**,**D**), *n* = 7–8; * *p* < 0.05; ** *p* < 0.01. (From [64], with permission).

Intriguingly, circulating levels of miR-153 and miR-223 were similarly lower in the 19-month-old GFAP.HMOX1^8.5−19 m^ mice compared to age-matched WT controls (Figure 3A,B) [64], commensurate with the systemic alterations in α-synuclein gene and protein expression profiles in GFAP.HMOX1^8.5−19 m^ mouse erythrocytes (Figure 3C,D), as well as in idiopathic human PD [15,18,55,64,93]. The peripheral changes in miR-153 and miR-223 levels in GFAP.HMOX1^8.5−19 m^ mice are what led to the study of these key miRNAs in human PD saliva as potential biomarkers of the disease.

## 5. miR-153, miR-223 and Heme Oxygenase-1 as Biomarkers of Parkinson Disease

Prior to evaluating miR-153 and miR-223 levels in human PD, we assessed the potential of HO-1 protein to serve as a biomarker for this condition. In 2018, we published a study reporting increased concentrations of salivary HO-1 in early stage (Hoehn and Yahr [H&Y] stage 1) PD patients compared to non-neurological (healthy) controls [21]. This corroborated previous findings of elevated HO-1 levels in the serum and plasma of PD patients [52,94,95]. Saliva offers advantages over other biofluids as its acquisition is non-invasive, inexpensive and requires minimal training of personnel [96,97,98]. In 2021, we published a study corroborating the significant elevation of salivary HO-1 in PD subjects compared to non-neurological control subjects and non-degenerative neurological controls (multiple sclerosis, epilepsy, essential tremor, stroke, nerve pain) though not compared to subjects with other neurodegenerative conditions (AD, mild cognitive impairment) [20]. Importantly, overexpression of HO-1 protein and mRNA has also been observed in AD and mild cognitive impairment [99,100,101]. Considering that HO-1 has also been linked to a variety of neurodegenerative conditions, including AD and mild cognitive impairment, the lack of specificity of high salivary HO-1 levels in PD is not surprising. Receiver operating characteristic (ROC) curves using HO-1 and covariates yielded areas under the curve above 85% in models for PD or neurodegenerative conditions relative to controls [20], suggesting HO-1 may be a reliable biomarker that distinguishes patients with PD from non-neurological and non-degenerative controls. Additional secondary analyses from the Galindez et al. study revealed a significant difference between non-PD controls and H&Y stage 2 PD participants [20]. More likely, HO-1 induction may be involved in the pathophysiology of neurodegeneration in general, rather than in one specific neurodegenerative condition. This may prove valuable in a clinical setting, as movement disorder specialists are often at a loss to differentiate idiopathic PD from neuroleptic-induced extrapyramidal disorders or vascular parkinsonism. Similarly, non-degenerative causes of mild cognitive impairment and dementia, such as toxic-metabolic encephalopathies or the psychomotor retardation complications of severe depression, may confound the diagnosis of AD. The ability to accurately distinguish between the degenerative and non-degenerative etiologies responsible for these clinical presentations by means of a salivary HO-1 protein or other simple biochemical assay, including miRNA analysis, would be a welcome development [20].

**Figure 4:**
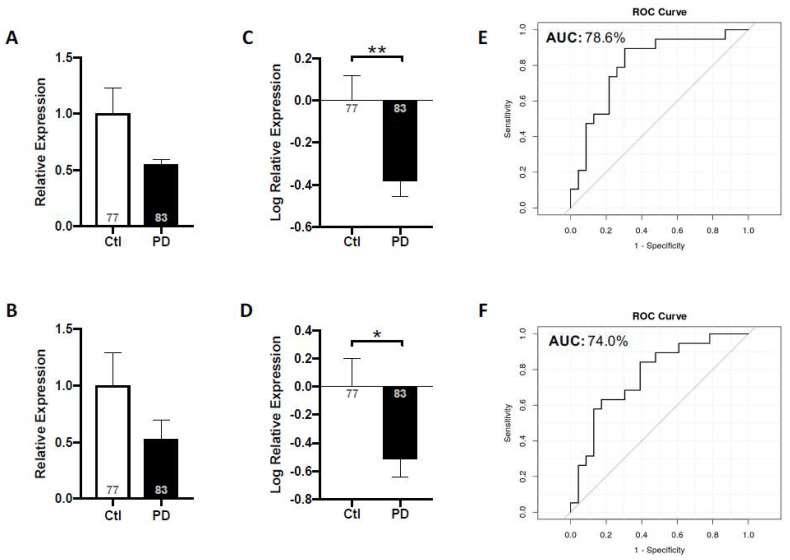
Salivary miR-153 and miR-223 expression levels in idiopathic PD subjects relative to non-neurological controls. Mean expression levels of miR-153 (**A**) and miR-223 (**B**) were determined by RT-qPCR and analyzed using the ΔΔCt method relative to internal and endogenous controls. Data was normalized via log-transformation and reported for miR-153 (**C**) and miR-223 (**D**). ROC curves were estimated for miR-153 (**E**) and miR-223 (**F**), with area under the curve indicated. Values shown within bars denote number of subjects. N = 77–83; * *p* < 0.05; ** *p* < 0.01. (From [19], with permission).

The results of our 2018 and 2021 studies on salivary HO-1 prompted us to measure miR-153 and miR-223, acting downstream of HO-1 and targeting α-synuclein, in PD saliva [19]. We observed that the expression levels of miR-153 and miR-223 were significantly reduced in PD saliva relative to that of non-neurological controls, and these expression patterns were unaffected by age, sex, various comorbidities, disease duration or medication exposure (Figure 4A–D) [19]. The area under the ROC curve separating controls from PD patients was 79% (confidence interval: 64–99%) for miR-153 and 74% (confidence interval: 60–93%) for miR-223 (Figure 4E,F) [19], suggesting moderately good biological markers of PD. Another miRNA that has been linked to α-synuclein regulation, miR-7 [85,102], was also assayed in saliva, though we did not observe significant alterations in miR-7a or miR-7b in PD patients relative to controls [19]. Additionally, the measurement of the ratios of key proteins acting upstream (HO-1) or downstream (oligomeric or total α-synuclein) of miR-153 or miR-223 to these miRNA expression levels did not improve the accuracy of the test as a PD neurodiagnostic relative to the ascertainment of miR-153 or miR-223 alone [19]. The alterations in salivary miRNAs described herein are consistent with our earlier findings in the brain and serum of parkinsonian GFAP.HMOX1^8.5−19 m^ mice [64].

MiR-153, miR-223 and *SNCA* expression levels in the human brain are highest in the midbrain, an important region at the epicenter of PD pathophysiology [85]. They have also been detected in extracellular compartments, including blood [93,103,104], CSF [15,105] and saliva [17,18,106], with their levels fluctuating in response to disease state. Several groups, in addition to our own, have linked miR-153 and, to a lesser extent, miR-223 to PD pathology [64,85,87,107]. Alterations in miR-153 levels have been documented in PD CSF [105] and plasma [108], while changes in miR-223 have been reported in PD serum [107]. To our knowledge, ours is the first study to document aberrant expression levels of miR-153 and miR-223 in the saliva of PD patients. However, considering other CNS conditions similarly involve differential expression of miR-153 and miR-223, it is critical to assay these miRNA levels in PD patients relative to neurological controls (e.g., other neurodegenerative conditions, other forms of parkinsonism, other synucleinopathies), using methodology akin to that of our 2021 salivary HO-1 study.

**Figure 5:**
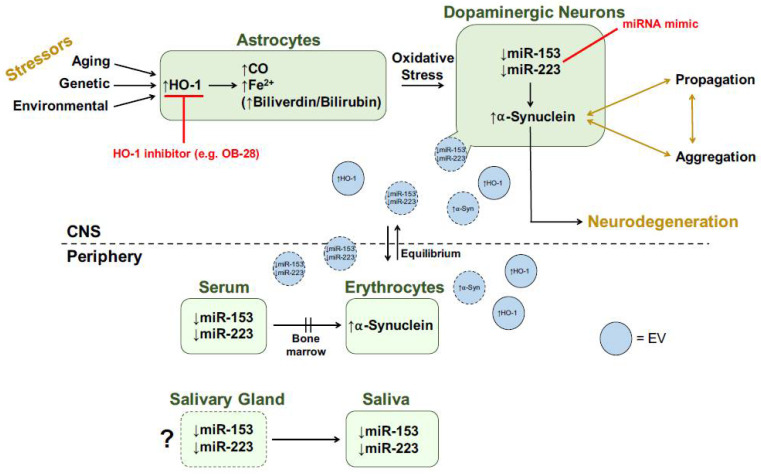
Transducer model for role of HO-1 in PD. Red text denotes potential therapeutic strategies. Shapes with dotted borders represent speculative hypotheses. See text for more details. CNS, central nervous system; CO, carbon monoxide; EV, extracellular vesicle; Fe^2+^, ferrous iron; HO-1, heme oxygenase-1; miRNA, microRNA; α-Syn, α-synuclein. Modified from [64].

It is unclear whether salivary HO-1, miR-153 and miR-223 originate as a transudate from plasma or are actively secreted by the salivary glands. In support of the latter, immunoreactive HO-1 has been demonstrated in normal salivary gland acini and duct epithelia and in benign salivary gland pleomorphic adenomas [109]. Salivary changes in HO-1 concentrations may also be associated mechanistically with the prominent autonomic innervation of the salivary glands and the relatively early appearance of Lewy pathology in the PD autonomic nervous system [14,110,111]. One possible mechanism of action for CNS changes being reflected in peripheral biofluids is the transfer of specific cargo across the blood–brain barrier (BBB) via extracellular vesicles (EVs). In fact, HO-1 protein, acting upstream of miR-153 and miR-223, was predominantly found in CNS-derived and non-CNS-derived EV fractions across five different human biofluids: saliva, plasma, serum, urine and CSF [112]. We hypothesize that CNS changes in key proteins (e.g., HO-1, α-synuclein) and miRNAs (e.g., miR-153, miR-223) are being reflected in the periphery via centrifugal EV transport (Figure 5).

The latter has important therapeutic and diagnostic implications for PD research. For instance, systemic exosomal siRNA delivery in transgenic mice expressing the human phosphorylation-mimic S129D α-synuclein (which exhibit aggregation pathology) significantly reduced levels of α-synuclein mRNA and protein as well as intracellular protein aggregates within dopaminergic neurons of the substantia nigra relative to WT controls [113]. While the therapeutic potential of EVs has not been fully elucidated in humans, their use holds promise for the treatment of different disorders of the CNS, where many drugs are of limited benefit due to their inability to penetrate the BBB [114]. The ability of EVs to cross the BBB via transcytosis has been experimentally confirmed [115,116,117]. Furthermore, EVs have been extensively studied in recent years as a potential source of biomarkers for PD, particularly neural-derived EVs, which offer a ‘window’ into the CNS. We are currently using nano-flow liquid-chromatography mass spectrometry to analyze differences, if any exist, between salivary EV and whole saliva proteomic profiles (Cressatti and Schipper, unpublished results). This line of inquiry should reveal whether EVs portray a more precise snapshot of PD pathophysiology than whole biofluids.

## 6. Concluding Remarks: HO-1, miR-153, miR-223 and α-Synuclein

In contrast to existing toxin and genetic animal models of PD, the GFAP.HMOX1^8.5−19 m^ mouse model of PD unveils a third category of animal model, namely the transducer model. GFAP.HMOX1^8.5−19 m^ mice recapitulate key features of the human disorder, such as non-regulated brain iron trapping, oxidative stress, mitochondrial damage and macroautophagy. These core pathological features common to neurodegeneration may represent a single lesion devolving from the sustained induction of *HMOX1* within astrocytes. This formulation strengthens the conceptual link between normal brain aging, often characterized by a milder version of this core pathology, and the major senescence-associated neurodegenerative disorders, such as PD [22]. For instance, when present in abundance, certain pathological features are considered characteristic of PD. This includes gliosis, increased α-synuclein protein, brain iron deposition and mitochondrial insufficiency [118]. However, when present at low densities, these are instead construed as features of normal brain aging [119,120]. Recognition that this core cytopathology drives the neurodegenerative process may shift the design of disease-modifying therapeutics towards the disruption of molecular pathways which act to integrate the constituents of this multifaceted lesion [22]. This highlights the astroglial HO-1 transducer as a potential therapeutic target to simultaneously mitigate several core pathological features of the disease.

Acting downstream of HO-1, miR-153 and miR-223 were identified as negative regulators of α-synuclein and moderately good diagnostic biomarkers of idiopathic PD [19,64]. The presence of these miRNA alterations in saliva is particularly relevant considering saliva acquisition is a minimally invasive and user-friendly protocol for the elderly PD population. Furthermore, peripheral changes in miR-153 and miR-223 were similarly observed in GFAP.HMOX1^8.5−19 m^ mouse and human PD brains, and EVs may offer a suitable mechanism of action to explain this phenomenon. It is important to note that this is not only relevant to miR-153 and miR-223 but also to other key players involved in PD neuropathology, including native and oligomeric α-synuclein, DJ-1, tau, other salient miRNAs and HO-1. Understanding how peripheral pathologies reflect CNS afflictions may accelerate our knowledge relating to CNS physiology in health and disease.

Most importantly, contemporary pharmacotherapy for PD is almost exclusively symptomatic in nature and effective neuroprotection would be a welcome development. Several metalloporphyrin inhibitors of HO activity are already in clinical use for the control of neonatal hyperbilirubinemia (jaundice) and certain adult liver conditions and, if secondary (‘bystander’) effects are successfully mitigated, could be adapted for the treatment of PD. The small-molecule inhibitor OB-28 may confer additional advantages for human use in light of its selectivity for HO-1 over HO-2, BBB permeability and favorable toxicity profile [60]. After many years of exploring the fundamentals of glial HO-1 behavior in CNS senescence and disease, we may now be at the cusp of translating this experience into the development of definitive, disease-modifying approaches to the management of PD and related neurodegenerative disorders—an unmet clinical need that heavily impacts the health and wellbeing of our aging population.
